# Association between transcriptomic metrics of exogenous antigen presentation and adaptive immunity with locoregional recurrence in localized estrogen receptor negative breast cancer: retrospective review of multi-institutional datasets

**DOI:** 10.1186/s13058-025-01987-x

**Published:** 2025-05-13

**Authors:** Timothy J. Robinson, Casey L. Liveringhouse, Christopher Wilson, Sam Friedman, Justyn Nakashima, Matthew N. Mills, Jacob D. Purcell, Nicholas B. Figura, Du Dongliang, Ram Thapa, Eric Welsh, Kamran A. Ahmed, G. Daniel Grass, Brooke L. Fridley, Roberto Diaz

**Affiliations:** 1 Department of Therapeutic Radiology, Yale School of Medicine, New Haven, CT USA; 2 Department of Radiation Oncology, H. Lee Moffitt Cancer Center and Research Institute, Tampa, FL USA; 3 Biostatistics and Bioinformatics Shared Resource, H. Lee Moffitt Cancer Center and Research Institute, Tampa, FL USA; 4 Michigan State University College of Human Medicine, East Lansing, MI USA

**Keywords:** Breast cancer, Estrogen receptor negative, Immune response, Locoregional control

## Abstract

**Background:**

Transcriptomic features of breast cancer locoregional recurrence (LRR) remain poorly understood. We therefore sought to investigate transcriptomic features associated with LRR in newly diagnosed invasive breast tumors from our institutional dataset.

**Methods:**

Transcriptomic profiling was performed on 632 tumors from consecutive patients treated within our health system for newly diagnosed non-metastatic breast cancer. Univariable Cox models identified genes whose expression was associated with LRR (*q*-value < 0.05). Up-regulated (UR) genes were defined as hazard ratio (HR) > 1 and down-regulated (DR) genes were defined as HR < 1. Gene set enrichment analyses were performed for UR and DR gene sets and validated within two external cohorts of ER- tumors.

**Results:**

With a median follow-up of 7.6 years, we observed 38 LRRs: 28/481 (5.8%) in ER + and 10/151 (6.6%) in ER-. There were 43 UR and 7 DR genes associated with LRR in ER + tumors, while 417 UR and 1150 DR genes were associated with LRR in ER- tumors. UR genes in ER + tumors were enriched for roles in cell proliferation (*q* < 0.05). In contrast, LRR in ER- tumors was most strongly associated with DR genes enriched for MHC-II-mediated antigen presentation and T cell activation (*q* < 0.05). In external cohorts of ER- tumors, 97 significant DR genes (*p* < 0.05) were enriched for 18 pathways, including 5 pathways involved in MHC-II signaling, antigen presentation and T-cell activation.

**Conclusions:**

Transcriptomic patterns associated with LRR appear distinct between ER + and ER- tumors. In ER + tumors, LRR appears predominantly associated with proliferation, whereas ER- LRR suggests a robust pattern of suppressed antigen presentation via MHC-II.

**Supplementary Information:**

The online version contains supplementary material available at 10.1186/s13058-025-01987-x.

## Background

While there are well-defined clinical risk factors for the development of locoregional recurrence (LRR) of breast cancer following definitive treatment, clinical outcomes cannot be accounted for by clinical factors alone [1, 2]. Molecular signatures have been developed to predict the risk of distant disease recurrence and now guide adjuvant systemic therapy in many patients with early-stage hormone receptor positive (HR +) breast cancer [3, 4]. Such signatures have also been retrospectively evaluated in secondary analyses of systemic therapy trials, demonstrating the ability to predict for LRR after standard local therapy in HR + and human epidermal growth factor receptor-2 negative (HER2-) disease (LN-) [5, 6]. While clinical trials utilizing genomic signatures to guide radiotherapy (RT) are ongoing, there is no standard of care genomic tool tailored to predict LRR or guide local management decisions. As a result, local (i.e. whole breast) or locoregional (i.e. whole breast plus adjacent nodal stations) RT remains the standard of care for women undergoing breast conserving therapy (BCT), despite the fact that only a subset of women benefit from treatment. Improved stratification of risk for LRR is therefore vital to identify women who may benefit from RT and spare women from RT in whom it is unnecessary.

Many genomic classifiers have been developed specifically to assess local recurrence risk or predict RT benefit [7–12], with mixed results on external validation, possibly owing to differences in technical factors or cohort composition, and limited availability of publicly available datasets with annotated local recurrence information. Further, these classifiers have focused largely on the estrogen receptor positive (ER +) subset of breast cancer, with relative under-representation of ER- tumors, and validation studies suggest these signatures may perform differently based on ER status [7, 9, 11]. Therefore, ER + and ER- tumors may necessitate separate genomic classifiers of LRR to reflect underlying differences in tumor biology. To our knowledge, no genomic classifier of LRR has been developed exclusively in the context of ER- breast cancer.

We posit that improved understanding of the biology of LRR with respect to breast tumor subtype may lead to (1) more robust genomic models to improve LRR stratification and RT treatment selection for localized breast cancer and (2) identify potential novel mechanisms by which to augment locoregional control. In this study, we utilized gene expression profiling to investigate transcriptomic factors associated with LRR in patients with ER + and ER- localized breast cancer a multi-institutional real-world cohort.

## Methods

### Patients

Primary breast tumor samples were identified from a prospective tissue collection protocol representing one academic and two community hospitals. Inclusion criteria included chemotherapy-naive women of any age with non-metastatic, locoregionally invasive breast carcinoma with known estrogen receptor status. Patients were treated consecutively, and all treatment decisions were according to provider and patient decision. Pathological and clinical information was collected by retrospective review of clinical charts.

### Transcriptomic profiling and intrinsic subtype classification

Gene expression analysis of tumor samples was performed with Affymetrix Rosetta/Merck Human HURSTA 2.0 microarray chips (Thermo Fisher Scientific, MA, USA). Expression values were normalized against the medial CEL file using IRON to provide logged intensity probeset values as previously reported as part of the Total Cancer Care institutional protocol [13]. Tumor molecular subtype was determined with SCMGENE [14] and the PAM50 [15] gene classifiers (R version 3.5.1, package genefu 2.14.0).

### Identification of expression of genes associated with time to LRR

Univariable and multivariable Cox proportional hazards models were fit to assess association of expression of each gene with time to LRR, and *p*-values were adjusted using the q-value method to minimize false discovery rate [16]. Genes with expression associated with time to LRR with a hazard ratio (HR) > 1 (i.e., increased gene expression associated with increased hazard for LRR) are defined as upregulated, and genes with expression associated with time to LRR and HR < 1 are defined as downregulated (i.e., increased gene expression associated with decreased hazard for LRR). This was performed separately for ER + , ER-, and HER2 + patients. All multivariable models included margin status and the multivariable model for HER2 + patients also included ER status. Additional factors such as T stage, N stage, and grade were not included in the models, as the goal of this study was to uncover the biology associated with LRR and we did not want to exclude genes just because they were associated with more aggressive disease. Cox models and FDR adjusted *p*-values were implemented using R version 4.4.1, package survival 2.42–3 and *q *value 2.14.0, respectively.

### Gene set enrichment analysis

Gene set enrichment analysis (GSEA) was performed separately for up- and downregulated genes with the Enrich R tool (http://amp.pharm.mssm.edu/Enrichr/) [17, 18]. Gene network analysis was performed with the IntAct database to identify co-expressed networks of genes with biological relevance (http://www.ebi.ac.uk/intact) [19].

### Network analysis

In order to gain further insight of the complex relationship between individual genes, a Gaussian graphical model (GGM) [20] was constructed on the probeset level. Gaussian graphical models (GGMs) measure the direct correlation between two probes. Direct correlation between any two probes is computed adjusting for all other probes. Once the direct correlation is computed for each pair of probes, A modified lasso regression procedure (53) is used in order to construct a sparse network, i.e. a network with a smaller number of edges as the penalty increases. Modules were identified by using Weighted Gene Co-expression Network Analysis (WCGNA) (16, 21), which uses hierarchical clustering to assign each gene to a module. Two genes are assigned to the same module if they share a large number of edges with other probes (neighbors). This analysis can be done in two ways: (1) the strength of the connection between two probes are assumed to be the same (unweighted), or (2) the strength of connections between the probes are allowed to vary (weighted). Since we have already conducted lasso to identify the significant edges, WCGNA will be conducted on an unweighted network. In this setting, the modules are interpreted exactly as previously described. Once probes are assigned to modules, we then identify hubs by computing the number of neighbors that each probe has within that corresponding module. The probe that has the most neighbors is considered to be the hub of that module. GGMs were implemented in R (version 3.5.1) using Genenet v1.2.13.

### Tumor infiltrating immune cell type deconvolution

We employed the CIBERSORT LM22 signature [21] to estimate the proportion of 22 types of tumor-infiltrating leukocytes (TILs). We used two sample t-tests to determine if there is difference in the mean proportion of immune cell types in patients who had or did not have LRR. Additionally, Cox proportional hazard models were performed to determine if any immune cell type in the CIBERSORT signature are related to risk LRR. Lastly, we examined the correlation between T-cell type proportions and gene expression from major histocompatibility complex (MHC)-II protein complex gene set. The CIBERSORT signature was implemented using CIBERSORT v1.0

### External validation

To determine if there are common themes in other studies to identify genes associated with risk of LRR, univariable Cox models were fit and GSEA was employed in two independent datasets accessed via the Gene Expression Omnibus (GEO) [8, 9]. The Servant dataset (GEO:GSE30682) is composed of 343 patients age < 50 with breast cancer treated with breast conserving surgery and adjuvant whole breast RT, including 76 ER- tumors [8]. The Sjostrom dataset (GEO:GSE103746) is composed of 172 patients treated with breast conserving surgery at six centers in Sweden, including 70 ER- tumors [9]. The genes included in the GSEA had a *p*-value < 0.05 on univariable Cox regression for LRR in their respective studies. All analyses in external cohorts were restricted to the ER- patients, and separate analyses are presented for genes with positive and negative association risk of LRR.

### Statistical analysis

Patient and clinical characteristics were summarized using descriptive statistics including median and range for continuous measures, and proportions and frequencies for categorical measures. Associations between continuous and categorical variables were evaluated using Kruskal–Wallis tests and means comparisons of continuous variables with T-tests where appropriate. The associations between categorical variables were evaluated using Chi-squared tests or Fisher’s exact tests where appropriate. Time-to-event analysis was conducted using the minimum of time from date of diagnosis to LRR with univariable Cox models and the Kaplan Meier method with log rank tests.

### Data availability

Gene expression data with matching clinical information including patient age, stage, receptor status, grade, treatment information and locoregional recurrence status are uploaded to the Gene Expression Omnibus (GSE199633) and can be accessed at https://www.ncbi.nlm.nih.gov/geo/query/acc.cgi?acc=GSE199633.

## Results

### Cohort characteristics

A total of 632 patients met inclusion criteria for analysis. Median follow-up from time of diagnosis was 7.6 years. The median age at time of diagnosis was 61 years (range 24–95), 76% of tumors were ER + , 91.6% were pT1-T2, and 40.5% had positive lymph nodes (Table 1). A total of 23.2% of patients were HER2 + and 30.5% were considered HER2 low [22] (Supplemental Tables A and B). We observed 38 LRRs: 28/481 (crude rate 5.8%) in ER + and 10/151 (crude rate 6.6%) in ER- tumors. Sixty-three patients (10%) experienced distant recurrence. Of this distant recurrence subset, 43 patients (68.3%) died. Of all 632 patients, 99 (15.7%) died.

**Table 1:** Patient characteristics

	Overall	ER-	ER +	*p* Value	No LRR	LRR	*p* Value
	*N* = 632	*n* = 151	*n* = 481		*n* = 516	*n* = 116	
Age at diagnosis (years, median)	61.0	56.7	62.2	< 0.001	59.5	67.9	< 0.001
Age at diagnosis (years, range)	24.9–95.5	25.6–89.3	24.9–95.5		24.9–94.5	25.3–95.5	
Histology				< 0.001			0.75
Invasive ductal	519 (82.1%)	145 (96.0%)	374 (77.8%)		423 (82.0%)	96 (82.8%)	
Invasive lobular	72 (11.4%)	2 (1.3%)	70 (14.6%)		58 (11.2%)	14 (12.1%)	
Other	41 (6.5%)	4 (2.6%)	37 (7.7%)		35 (6.8%)	6 (5.2%)	
PR Positive	399 (63.2%)	9 (6.0%)	390 (81.0%)	< 0.001	334 (64.7%)	65 (56.0%)	0.088
HER2 Positive	111 (17.6%)	41 (27.2%)	70 (14.6%)	0.001	93 (18.0%)	18 (15.5%)	0.45
pT stage				0.095			< 0.001
1	312 (49.4%)	76 (50.3%)	236 (49.1%)		275 (53.3%)	37 (31.9%)	
2	166 (42.1%)	70 (46.4%)	196 (40.7%)		206 (39.9%)	60 (51.7%)	
3	44 (7.0%)	3 (2.0%)	41 (8.5%)		30 (5.8%)	14 (12.1%)	
4	7 (1.1%)	1 (0.7%)	7 (1.1%)		3 (0.6%)	4 (3.4%)	
1mi	2 (0.3%)	1 (0.7%)	1 (1.1%)		1 (0.2%)	1 (0.9%)	
Unknown	1 (0.2%)	0	1 (0.2%)		1 (0.2%)	0	
Lymph node positive	256 (40.5%)	51 (33.8%)	205 (42.6%)				
pN Stage				0.14			0.001
0	366 (58.0%)	99 (66.0%)	267 (55.5%)		305 (59.1%)	61 (52.6%)	
1	152 (24.1%)	32 (21.3%)	120 (24.9%)		126 (24.4%)	26 (22.4%)	
2	50 (7.9%)	11 (7.3%)	39 (8.1%)		36 (7.0%)	14 (12.1%)	
3	25 (4.0%)	3 (2.0%)	22 (4.6%)		15 (2.9%)	10 (8.6%)	
1mi	29 (4.6%)	5 (3.3%)	24 (5.0%)		28 (5.4%)	1 (0.9%)	
Unknown	10 (1.6%)	1 (0.7%)	9 (1.9%)		6 (1.2%)	4 (3.4%)	
Local therapy				0.33			0.069
Mastectomy alone	232 (36.7%)	64 (42.4%)	168 (34.9%)		194 (37.6%)	38 (32.8%)	
Mastectomy + radiation	120 (19.0%)	22 (14.6%)	98 (20.4%)		95 (18.4%)	25 (21.6%)	
Lumpectomy alone	17 (2.7%)	4 (2.6%)	13 (2.7%)		10 (1.9%)	7 (6.0%)	
Lumpectomy + radiation	254 (40.2%)	60 (39.7%)	194 (40.3%)		211 (40.9%)	43 (37.1%)	
Unknown	9 (1.4%)	1 (0.7%)	8 (1.7%)		6 (1.2%)	3 (2.6%)	
Positive Surgical Margins	34 (5.4%)	8 (5.3%)	26 (5.4%)	0.85	21 (4.1%)	13 (11.2%)	0.008
Received adjuvant chemotherapy	363 (57.4%)	124 (82.1%)	239 (49.7%)	< 0.001	312 (60.5%)	51 (44.0%)	0.004
Pam50 Molecular Subtype				< 0.001			0.16
Basal	82 (13.0%)	74 (49.0%)	8 (1.7%)		64 (12.4%)	18 (15.5%)	
Her2	76 (12.0%)	46 (30.5%)	30 (6.2%)		61 (11.8%)	15 (12.9%)	
LuminalB	179 (28.3%)	6 (4.0%)	173 (36.0%)		228 (44.2%)	38 (32.8%)	
LuminalA	266 (42.1%)	15 (9.9%)	251 (52.2%)		138 (26.7%)	41 (35.3%)	
Normal	29 (4.6%)	10 (6.6%)	19 (4.0%)		25 (4.8%)	4 (3.4%)	
SCMGENE Molecular Subtype				< 0.001			0.47
ER-/HER2-	110 (17.4%)	90 (59.6%)	20 (4.2%)		88 (17.1%)	22 (19.0%)	
ER + /HER2- High prolif	288 (45.6%)	18 (11.9%)	270 (56.1%)		230 (44.6%)	58 (50.0%)	
ER + /HER2- Low prolif	160 (25.3%)	6 (4.0%)	154 (32.0%)		137 (26.6%)	23 (19.8%)	
HER2 +	74 (11.7%)	37 (24.5%)	37 (7.7%)		61 (11.8%)	13 (11.2%)	

*ER*, Estrogen receptor; *PR*, Progesterone receptor; *HER2*, Human epidermal growth factor receptor 2; *LRR*, Locoregional recurrence

### Analysis of LRR reveals distinct genomic pathways of tumor progression

**Fig. 1:**
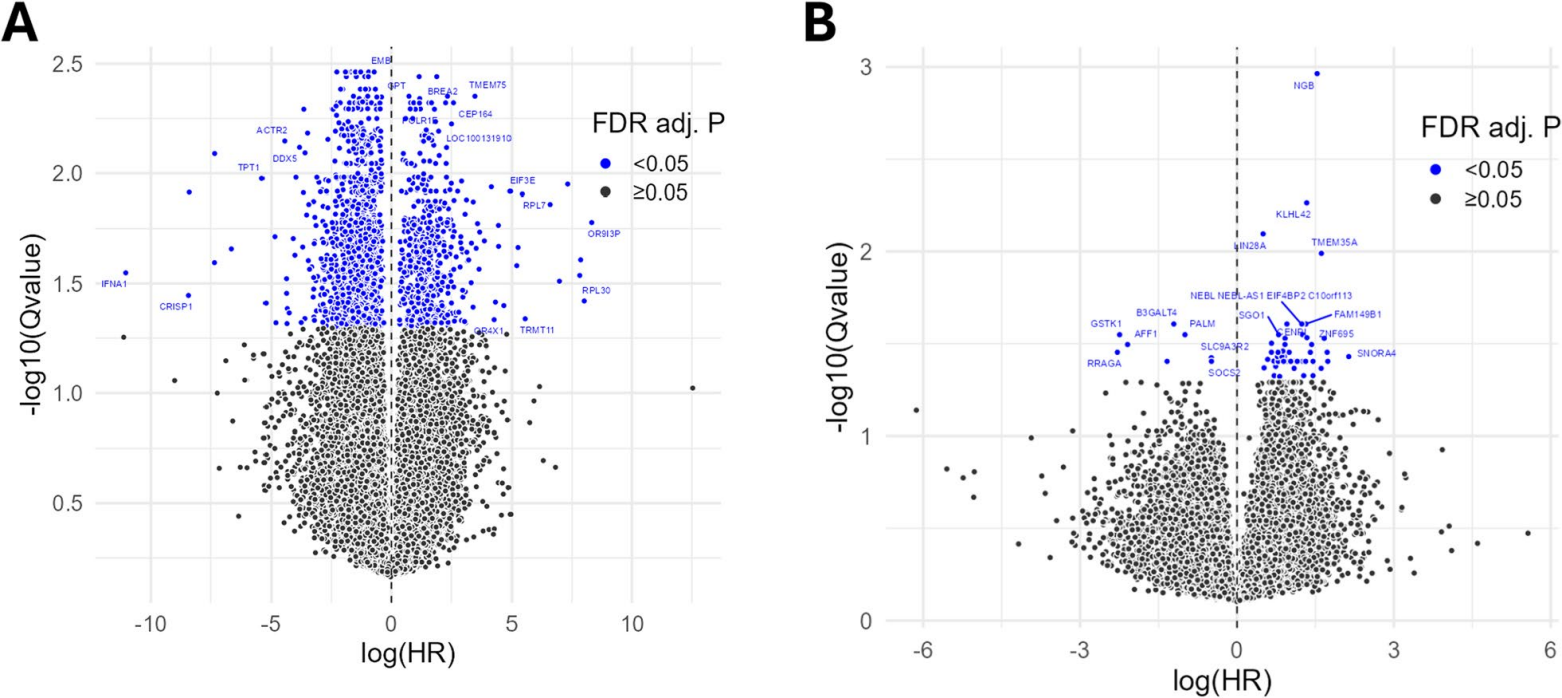
Volcano plots displaying results of univariate Cox regression relating gene expression to time to locoregional recurrence for subsets of ER- (**A**) and ER + (**B**) tumors. In each panel, genes with an FDR-adjusted *p*-value less than 0.05 are colored blue. *p*-values were FDR-adjusted using Benjamini–Hochberg procedure

Univariable Cox regression identified 50 genes associated with time to LRR in ER + tumors and 1567 genes associated with time to LRR in ER- tumors. (*q* < 0.05) (Supplemental Table 2). In ER- tumors, 417 genes were up-regulated and 1150 were down-regulated in their association with LRR (Fig. 1A), suggesting that inhibitory (i.e. down-regulation) transcriptional events were more strongly correlated with LRR than activating events (i.e. up-regulation). There were only four overlapping genes associated with LRR in both ER + and ER- cohorts. In ER + tumors, this included 43 genes that were up-regulated and 7 genes that were down-regulated in their association with time to LRR (Fig. 1B). The univariable Cox regression showed no significant genes in the HER2 + cohort**.**

In the multivariate case of Cox regression, in which margin status was also included as a covariate, we identified 97 genes associated with time to LRR in ER + tumors and 1794 genes associated with time to LRR in ER- tumors (*q* < 0.05). In ER + tumors, this included 82 genes that were up-regulated (gene expression HR > 1) and 15 genes that were down-regulated (HR < 1). In ER- tumors, 468 genes were up-regulated and 1327 were down-regulated in their association with LRR, showing that the correlation between inhibitory transcriptional events and LRR is maintained when margin status is considered in the model. The multivariate Cox model also analyzed samples of HER + tumors, in which 217 genes were found to be associated with time to LRR. Of these genes, 54 were up-regulated and 163 were down-regulated, showing a similar trend of correlation as seen in the ER- cohort.

GSEA identified distinct roles for genes associated with LRR in both ER- and ER + tumors. Up-regulated genes associated with LRR in ER + tumors were notable for roles in the centromere complex during cell division, which was confirmed by enriched biological pathways. While we identified 72 pathways significantly associated with the down-regulated gene set in ER + tumors, each pathway had only one overlapping gene (Supplemental Table 3).

In contrast to ER + tumors, ER- tumors exhibited a far more robust transcriptome profile associated with LRR, particularly in the down-regulated gene set. In ER- tumors, GSEA identified 8 pathways associated with the up-regulated gene set, and 75 pathways associated with the down-regulated gene set (Supplemental Table 4). The set of up-regulated genes was enriched for roles in ribosomal and translational processes. The down-regulated gene set was enriched for roles in the immune response, as 33 of the 75 significant pathways were involved in generation of the adaptive immune response. Specifically and most notably, these immune response genes were enriched for roles in the exogenous antigen presentation pathway via antigen presentation on MHC-II and subsequent downstream adaptive T cell response via cytokine and interferon gamma (IFN*γ)* signaling, and B cell activation. Given existing genomic signatures of LRR in ER + tumors and the much stronger signal observed in ER- tumors, we subsequently focused our analyses on ER- tumors.

**Fig. 2:**
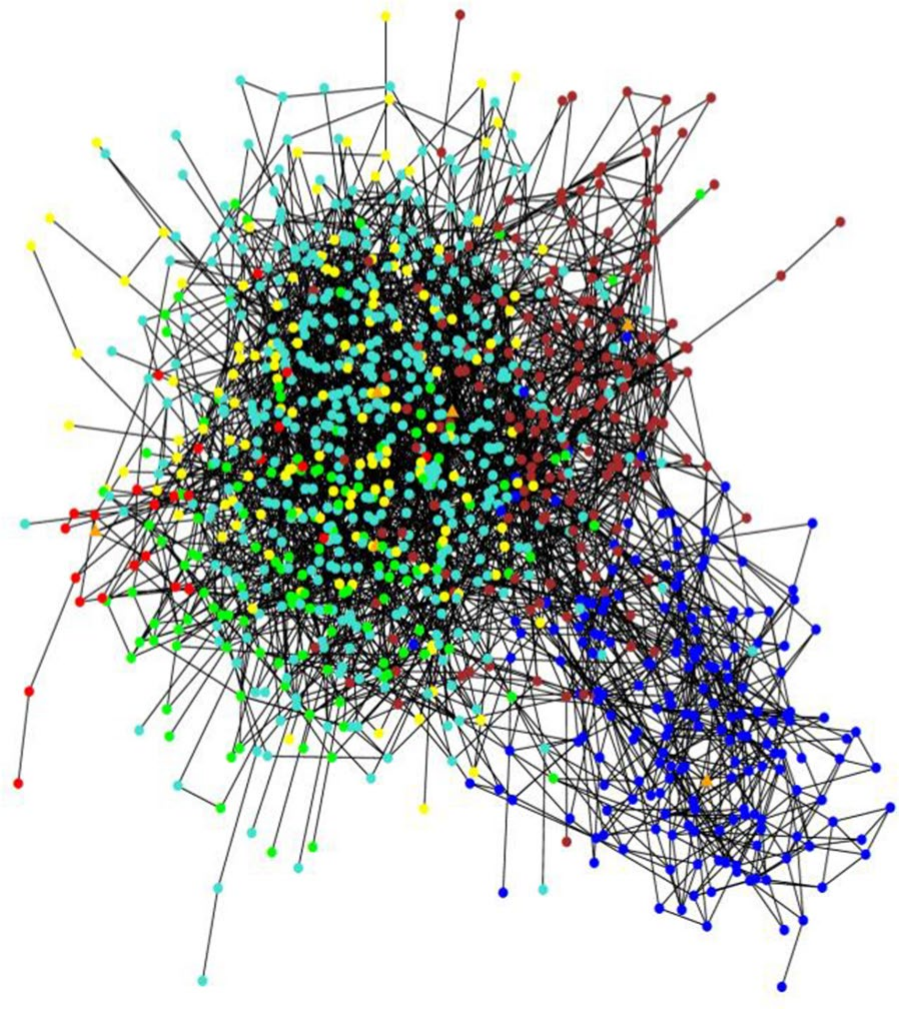
Visualization of gaussian graphical model for down-regulated gene set for ER- tumors. Circles represent individual genes. Each color represents membership in one of six modules. Lines represent “edges” or connections between genes. Module hub genes are those with the highest number of edges per module and are denoted with triangles. GSEA was used to assess the underlying biology in each module, where four of the six modules contained significantly enriched pathways loosely corresponding to MHC II signaling (blue), DNA damage response (turquoise), extracellular matrix organization (brown), and transcription regulation (red), respectively

Network modeling of ER- tumors reveals down-regulation of distinct immune modules associated with LRR:

We next performed Gaussian graphical modeling of the down-regulated genes from the ER- gene set and identified 6 network clusters optimized for co-expression (Fig. 2). GSEA was used to assess the underlying biology in each cluster, where four of the six clusters contained significantly enriched pathways (Supplemental Table 5). Most notably, the tightly clustered blue module contains genes enriched for immune activation pathways described above, including MHC-II and subsequent downstream adaptive T-cell and B-cell response signaling pathways. These findings identify the tumor antigen presentation process as a component of tumor biology with a robust role in the development of LRR.

### Individual gene-level analysis confirms MHC-II pathway downregulation as a primary feature of LRR

**Fig. 3:**
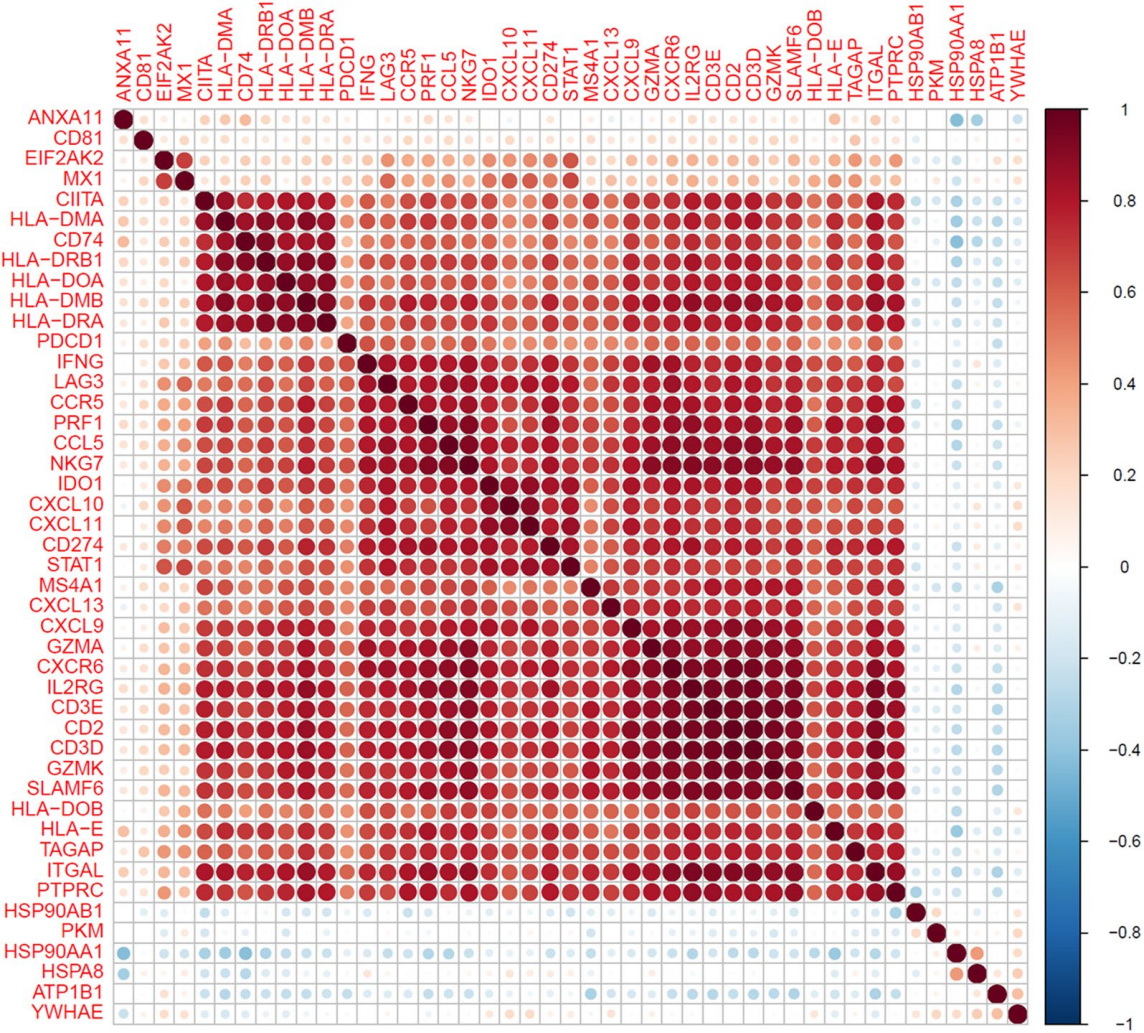
Correlation matrix of expression of down-regulated immune function genes in ER- tumors. Spearman correlation coefficients are denoted by circle size and color (red = positive, blue = negative, white/no circle = zero)

Structural MHC-II genes such as *CD74, HLA-DMA, HLA-DMB, HLA-DRA, HLA-DRB, HLA-DQA1*, *HLA-DPA1*, and *HLA-DOA* were significantly down-regulated, but highly co-expressed with strong correlation with expression of *CIITA*, the principal regulator of MHC-II expression, and *IFNγ*, a modulator of CIITA-induced MHC-II expression [23]. Likewise, there was also association between expression of MHC-II genes and *MX1* and *EIF2AK2*, known mediators of *IFNγ* [24, 25]. Expression of MHC-II genes were positively correlated with expression of downstream adaptive immune response genes including *IFNγ* and IFNγ-responsive chemokines (*CXCL9-11*) and signal transducers (*STAT1*), Th response (*LAG3*, *CD3*), B-cell function (*MS4A1*), and cytotoxic CD8 + T- and NK cell (NKC) function (*GZMA*, *GZMK*, *PRF1*, *NKG7*). Interestingly, there was also correlation between MHC-II and *CD274* (PD1) and *PCD1* (PDL-1) (Fig. 3).

Greater than median expression (“high expression”) of MHC-II genes such as *HLA-DQA1*, *HLA-DPA1*, *HLA-DOA* were each individually associated with improved locoregional control on KM survival analysis using the log-rank test (*p* < 0.05).

### CIBERSORT analysis identifies discrete TIL profiles associated with LRR

**Fig. 4:**
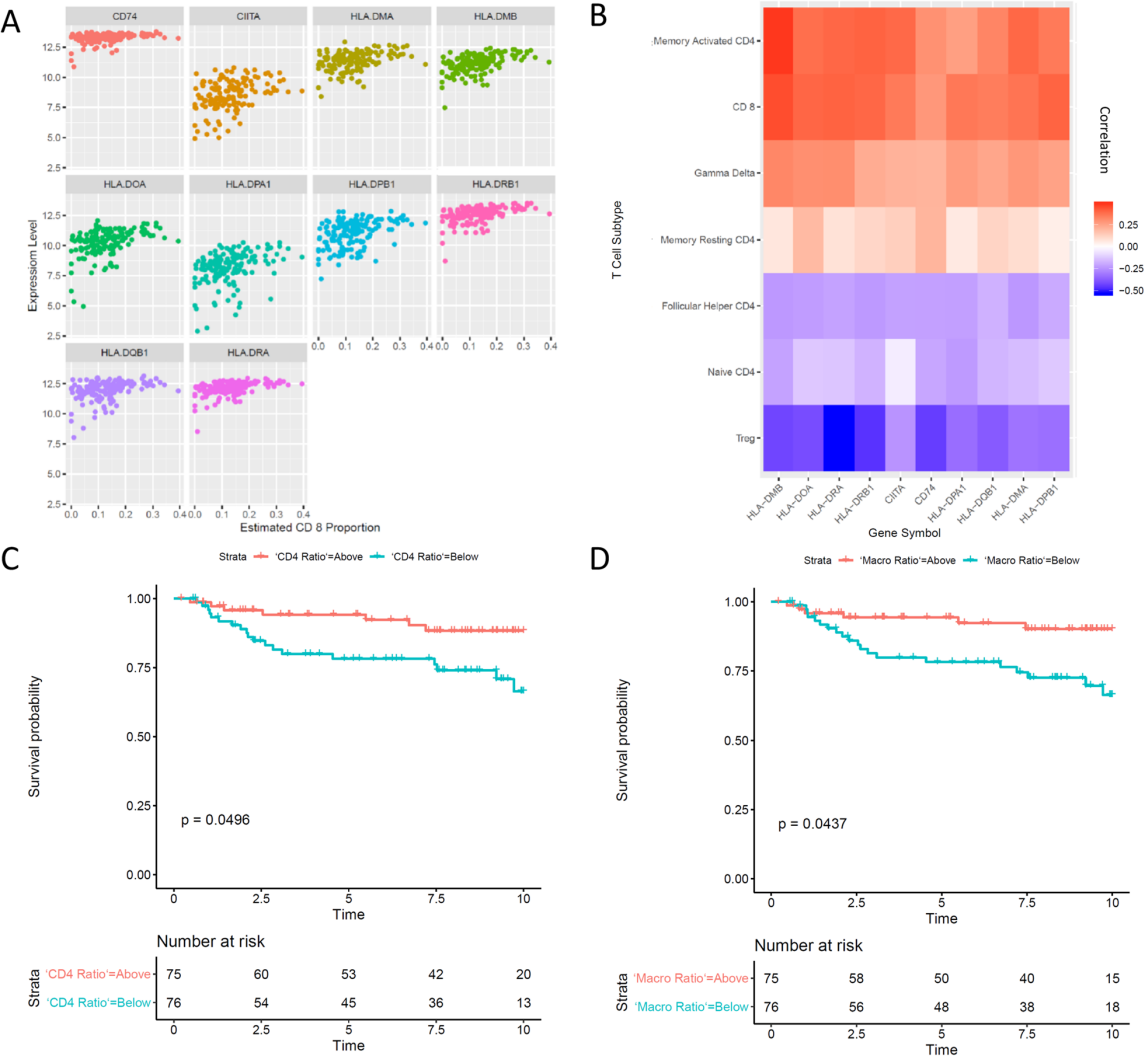
**A** Scatterplot of expression of CD74, CIITA and MHC-II HLA genes vs estimated CD8 + T cell proportion by CIBERSORT. **B** Heatmap of expression of CD74, CIITA, and MHC-II HLA genes vs proportion of T cell populations by CIBERSORT. Spearman correlation coefficients are denoted by 24 cell color (red = positive, blue = negative, white = zero). **C** Kaplan Meier plot comparing time to locoregional recurrence of activated CD4 + T cell to T reg ratio above cohort median (“CD4 Ratio = above”) to below the cohort median (“CD4 Ratio = below”). **D** Kaplan Meier plot comparing time to locoregional recurrence of M1 to M2 macrophage ratio above cohort median (“Macro Ratio = above”) to below the cohort median (“Macro Ratio = below”)

Given the suggestion that MHC II-based antigen presentation was downregulated and associated with LRR, we next investigated metrics of tumor immune cell infiltrates. Expression of MHC-II genes including HLA genes, *CIITA*, and *CD74* were associated with an anti-tumor T cell response, represented by positive correlation with proportion of infiltrating CD8 + T cells (Fig. 4A), and negative correlation with infiltration of immunosuppressive T regs (Fig. 4B**)**. MHC-II gene expression was also positively correlated (*p* < 0.05) with proportion of Th cells, and Gamma Delta T cells, and negatively correlated with naïve CD4 + T cells and T regs (*p* < 0.05) (Fig. 4C**, **Supplemental Table 6). While *HLA-DOB* and *HLA-DQA1* are included in the CIBERSORT LM22 gene matrix, they are not a dominant component of T cell signatures in the module [26]. There was weak association between MHC-II expression and proportion of resting and activated dendritic cells (DCs) for most MHC-II associated molecules; only *CIITA*, *HLA-DMA*, *-DMB*, and *DQA1* were weakly correlated with resting DCs, and only *CD74* was weakly correlated with activated DCs (*p* < 0.05) (Supplemental Table 6). *HLA-DOA* was positively correlated with the ratio of activated memory CD4 + T helper cells (Th) to T regs and the ratio of M1 macrophages to M2 macrophages, and weakly negatively correlated with the ratio of activated to resting DCs (Fig. 4D). An elevated ratio of Th to T regs and M1 to M2 macrophages above the cohort median value were each associated with decreased LRR on Kaplan Meier analysis (Fig. 4E, F), as was high proportion (greater than cohort median) of CD8 + T cells, activated NKCs, and memory B cells (*p* < 0.05).

### External validation shows conservation of down-regulated MHC-II pathways across cohorts

We next sought to validate the observed association between MHC-II immune inhibition and LRR using external cohorts. Assessment of gene expression from ER- tumors in two external datasets with LRR status (Servant *n* = 76, Sjostrom *n* = 70) identified a set of 97 genes associated with time to LRR (*q* < 0.05) replicated with the same directionality in each external cohort GSEA were performed for the up- and down-regulated gene sets in each cohort, utilizing a less stringent confirmatory (vs. exploratory) cutoff for gene inclusion (*p* < 0.01). There were no enriched pathways associated with the up-regulated gene sets in either external cohort. For the down-regulated gene sets, 18 enriched pathways from the primary analysis were replicated in both external cohorts, which mirrored our observation within the primary study cohort of down-regulation being the predominant transcriptional feature associated with LRR. Five of the 18 replicated pathways were associated with antigen processing and presentation and subsequent interferon gamma signaling and T cell receptor activation (Supplemental Table 7).

## Discussion

To our knowledge, we present the largest cohort to date of consecutively treated breast cancer patients with locoregional failure status and available genomic profiling. We identified a robust network of down-regulated genes in localized ER- breast cancer that are implicated in functions across the immune response spectrum, most notably for roles in antigen presentation through the MHC-II pathway. We identified an integrated network of adaptive immune response genes encompassing *IFNγ*-induced, *CIITA*-mediated MHC-II expression with subsequent infiltration and response of effector cells including CD4 + T helper, CD8 + T cells, and CD20 + B cells, all of which were associated with improved locoregional breast cancer control. MHC-II expression was correlated with a decreased proportion of pro-tumorigenic immune cells such as T regulatory cells and M2 macrophages. In the absence of association between MHC-II expression and a dendritic cell signature, these data suggest that it may be breast tumor cells themselves, rather than invading antigen presenting cells, that may be expressing these MHC-II markers. We then validated the association of down-regulated MHC-II pathways in ER- breast cancer with locoregional control in two external datasets. This population-level characterization of the biology of locoregional recurrence carries ramifications for risk stratification, treatment selection, and development of potential novel therapeutic strategies in ER- breast cancer.

There is an accumulating body of evidence linking the immune system with breast cancer outcomes and response to treatment. Triple negative breast cancer (TNBC) and ER-/HER2 + tumors are thought to be the most immunogenic breast cancer subtypes, with higher rates of immune cell infiltrates than their ER + counterparts [27]. Studies evaluating immune response in ER- breast cancers have identified distinct immunophenotypes associated with differing outcomes, and this has been proposed as a method to differentiate between high and low risk groups [28–30]. Further characterization of breast tumor immunobiology may provide insight into the mechanisms of immune escape and local failure, with these insights being incorporated into prediction models aiming to classify risk and improve treatment selection. Such strategies are sorely needed for ER- negative breast cancers, given the relatively limited representation of ER- tumors in previously published genomic signatures of local or regional recurrence.

Results from our study support the concept of the existence of contrasting immune-rich vs. immune-poor breast cancer subgroups, particularly in TNBC. Lehmann et al. described six distinct TNBC subtypes, including an immunomodulatory (IM) subtype with a molecular profile most notable for increased expression of genes encoding immune markers and signaling transduction, predominantly in antigen presentation and T cell effector processes [31]. Similarly, Bonsang-Kitzis et al. identified six distinct TNBC metagenes, including two immune-signature rich clusters, enriched for reflective of interferon alpha and gamma pathways (Immunity1), and B-, T- and CD8 + cell signatures (Immunity2) [32]. These results were further supported by Jezequel et al. who described two subsets of basal-like tumors with opposing immune infiltration profiles consisting of one group with elevated ratios of M2 to M1 macrophages and TGFβ signaling, in contrast to another group with signatures for a robust adaptive immune response with infiltration of T-cells, B-cells and activated type I interferon, MHC-I and MHC-II signaling features [33]. Thus, tumors with profiles for immune activation represent a distinct subgroup.

Our results suggest that the tumor subset with immune down-regulation, particularly in genes involved in antigen presentation via the MHC-II pathway, is associated with worse locoregional disease control, and to our knowledge marks the first study to report such an association. A low Immunity2 score in the Bonsang-Kitzis subtype classification was associated with poor disease-free survival [32], and Lehmann noted that IM classification was associated with improved prognosis regardless of TNBC subtype [34]. Forero et al. also reported the importance of antigen-presentation pathways, as they observed that up-regulation of MHC-II gene expression is highly associated with improved progression-free survival in triple negative cases [35]. Tumor characteristics and treatment profiles in the Lehman cohort were similar to ours, though their cohort was selected to have a relapse rate of 46.8%, whereas our cohort was consecutively treated and thus possibly more representative of the risk of LRR seen in practice. Importantly, the genes identified as among the most associated with progression-free survival by Forero include *CIITA*, *CD74*, and multiple *HLA-DP*, *-DQ*, and *-DR genes*, the same genes identified among the most important in our analysis. Similar results were reported by Ascierto et al., who observed that up-regulated genes associated with improved relapse free survival were enriched for concepts in antigen processing and presentation, and interferon gamma signaling [36]. We demonstrate these findings in multi-institutional and multi-national cohorts, suggesting a generalized conservation of this phenomenon across the ER- breast cancer population.

Prognostic gene signatures are widely used to predict disease-free survival in early-stage ER + tumors [3, 4]. Although signatures for LRR have also been developed in the research setting [9, 11, 37, 38], ER- tumors compose the minority in these cohorts. Recently-developed signatures aiming to predict intrinsic radiosensitivity such as the radiosensitivity signature (RSS) developed by Speers et al. at the University of Michigan [11] and the radiosensitivity index (RSI) developed by Torres-Roca et al. at Moffitt Cancer Center [37] showed disparate predictive and prognostic utility in ER + vs ER- tumors in an independent cohort [9]. With only 4 overlapping significant genes between ER + and ER- tumors and disparate results in pathway analyses, our results confirm differences in biology between ER + and ER- tumors with respect to locoregional recurrence and demonstrate the need for development of separate classifiers between the two subsets for optimal results. We posit that gene expression signatures attempting to stratify risk for LRR in ER- breast cancer should be ER-status specific, as signatures with an underlying coherent function may perform more reliably than those assembled with no regard for biology [39].

Recent evidence, including the randomized phase III KEYNOTE 522 study, demonstrates an increased rate of pathological complete response (pCR) and improved event-free survival with the addition of pembrolizumab, an anti-programmed death ligand-1 (PD-L1) monoclonal antibody, to standard neoadjuvant chemotherapy (NAC) for TNBC [40–42]. While these results are promising, patients receiving pembrolizumab had increased risk for severe adverse events, highlighting the importance of appropriate patient selection for anti-PD-L1 agents. While patients in KEYNOTE-522 with high tumor PD-1 staining received comparatively higher benefit from pembrolizumab, patients with low PD-1 also benefited. Thus, alternative biomarkers to PD-1 should be investigated in this regard to optimize patient selection criteria. Work by Johnson et al. demonstrated in two independent cohorts of melanoma patients treated with anti-PD-1 therapy, MHC-II positivity on tumor cells was associated with therapeutic response [43]. While breast tumor biology remains distinct from that of melanoma, we hypothesize that the presence of MHC-II expression might inform therapeutic selection for anti-PD1 and -L1 therapies.

There is increasing interest in novel strategies incorporating neoadjuvant radiotherapy to expose neoantigens and augment the anti-tumor immune response to overcome intrinsic resistance to immune checkpoint inhibitor therapy in TNBC [44]. While early studies in metastatic TNBC including the phase 2 TONIC trial from Netherlands Cancer Institute [45] and a multi-institutional phase 2 study [46] demonstrated somewhat disappointing overall response rates with combination RT and immunotherapy, our observations demonstrating the role of antigen presentation and subsequent downstream response in locoregional control suggest that the combination of locally ablative radiotherapy to augment systemic immune responses may be a rational approach for localized disease. We propose that a framework utilizing transcriptomic metrics of MHC-II/exogenous antigen presentation for selection of breast tumors which may harbor optimal biology to benefit from the combination of radiotherapy and immunotherapy and may inform future clinical trial design for appropriate integration of immunotherapy into the breast cancer treatment paradigm.

In the modern era, the standard of care for many ER- patients is treatment with NAC with a growing role for integration of neoadjuvant immune checkpoint inhibition in this setting as well. There is a rich literature investigating the immune response in TNBC and HER2 + breast cancer following NAC, demonstrating the correlation between immune infiltration and disease outcomes [47, 48] with respect to distant disease failure, which has not to our knowledge been reported with respect to LRR. Our study excluded patients treated with NAC due to the possibility that chemotherapy may alter gene expression, and thus, insights taken from the setting of NAC are limited. Similarly, the results described here require validation by tissue examination with immunohistochemical staining to elucidate the degree of tumor immune cell infiltration as well as the spatial distribution of these markers to support the hypothesized mechanism of immune activation. Future research is warranted to confirm proteomic-level and cell lineage (i.e. cancer vs. stromal) differences in MHC-II axis signaling.

## Conclusion

We provide evidence supporting the existence of distinct biologic pathways contributing to locoregional recurrence in ER + and ER- breast cancer subtypes. Consistent with previous literature, we observed that LRR in ER + tumors is associated with transcriptomic enrichment of genes with roles in proliferation. In contrast, ERR LRR was strongly correlated with transcriptomic down-regulation of MHC-II expression, antigen processing and presentation, and T-cell receptor signaling. We confirmed these findings in two independent cohorts, suggesting that immune-mediated suppression of LRR is a generalized feature of ER- tumors, but not necessarily ER + tumors. Our findings are consistent with the rapidly advancing understanding that the immune response has a prominent role in promoting optimal locoregional control in ER- breast cancer. Future investigation is warranted to understand the gene regulatory and therapeutic implications of this phenomena and may inform the rational combination of radiation therapy with immune-based therapeutics in the clinic.

## Supplementary Information

Additional file 1.

Additional file 2.

Additional file 3.

Additional file 4.

Additional file 5.

Additional file 6.

Additional file 7.

Additional file 8.

Additional file 9.

## Data Availability

The datasets generated and/or analyzed from our institution during the current study are available in the Gene Expression Omnibus repository (GSE199633) and can be accessed at https://www.ncbi.nlm.nih.gov/geo/query/acc.cgi?acc=GSE199633. Data from publicly available cohorts: Sjostrom et al. (GEO:GSE103746) can be accessed at https://www.ncbi.nlm.nih.gov/geo/query/acc.cgi?acc=GSE103746. Servant et al. (GSE30682) can be accessed at https://www.ncbi.nlm.nih.gov/geo/query/acc.cgi?acc=GSE30682.

## Abbreviations

LRR: Locoregional recurrence
HR +: Hormone receptor positive
HER2: Human epidermal growth factor receptor-2
RT: Radiotherapy
BCT: Breast conserving therapy
ER: Estrogen receptor
HR: Hazard ratio
GSEA: Gene set enrichment analysis
GGM: Gaussian graphical model
WGCNA: Weighted gene co-expression network analysis
TOM: Topographical overlap measure
TIL: Tumor infiltrating leukocyte
MHC-II: Major histocompatibility complex-II
GEO: Gene expression omnibus
IFNγ: Interferon gamma
TNBC: Triple negative breast cancer
IM: Immunomodulatory
RSS: Radiosensitivity signature
RSI: Radiosensitivity index
pCR: Pathologic complete response
PD-L1: Programmed death ligand-1
NAC: Neoadjuvant chemotherapy
UR: Up-regulated
DR: Down-regulated
