# Supplementary material for: Association between transcriptomic metrics of exogenous antigen presentation and adaptive immunity with locoregional recurrence in localized estrogen receptor negative breast cancer: retrospective review of multi-institutional datasets
Source: Breast Cancer Res. 2025 May 13;27:77. doi: 10.1186/s13058-025-01987-x (PMC12070507; doi:10.1186/s13058-025-01987-x)
Supplement: Supplementary file 2 — Additional file 2. [file 13058_2025_1987_MOESM2_ESM.docx]

HER2 data

| **IHC** |  |
| --- | --- |
| Not performed/Unknown | 70 |
| 0 or negative | 222 |
| 1+ | 189 |
| 2+ | 81 |
| 3+ | 75 |
| **FISH** |  |
| Negative or 0 | 9 |
| Positive | 334 |
| Not performed/Unknown | 294 |
| **HER2 Status** |  |
| HER2 Zero | 222 |
| HER2 Low | 194 |
| HER2 Positive | 148 |
| Unknown | 73 |

HER2 status defined as described in [HER2-Low Breast Cancer: Pathological and Clinical Landscape | Journal of Clinical Oncology](https://ascopubs.org/doi/10.1200/JCO.19.02488?url_ver=Z39.88-2003&rfr_id=ori:rid:crossref.org&rfr_dat=cr_pub%20%200pubmed)
